# Relationship between Eating Disturbance and Dementia Severity in Patients with Alzheimer’s Disease

**DOI:** 10.1371/journal.pone.0133666

**Published:** 2015-08-12

**Authors:** Kyoko Kai, Mamoru Hashimoto, Koichiro Amano, Hibiki Tanaka, Ryuji Fukuhara, Manabu Ikeda

**Affiliations:** 1 Department of Neuropsychiatry, Graduate School of Medical Science, Kumamoto University, Kumamoto, Japan; 2 Department of Neuropsychiatry, Faculty of Life Sciences, Kumamoto University, Kumamoto, Japan; 3 Department of Psychiatry, Nozaki Hospital, Miyazaki, Japan; Nathan Kline Institute and New York University School of Medicine, UNITED STATES

## Abstract

**Background:**

Eating is one of the most important daily activities in managing patients with dementia. Although various eating disturbance occur as dementia progresses, to our knowledge, most of the studies focused on a part of eating disturbance such as swallowing and appetite. There have been few comprehensive studies including eating habits and food preference in patients with Alzheimer’s disease (AD). The aims of this study were to investigate almost all eating disturbance and to examine the relationship of eating disturbance to dementia stage in AD.

**Methods:**

A total of 220 patients with AD and 30 normal elderly (NE) subjects were recruited. Eating disturbance was assessed by a comprehensive questionnaire that had been previously validated. Potential relationships between the characteristics of eating disturbance and dementia stage as classified by the Clinical Dementia Rating (CDR) were assessed.

**Results:**

Overall, 81.4% of patients with AD showed some eating and swallowing disturbance, whereas only 26.7% of the NE subjects had such a disturbance. Even in an early stage, patients with AD had many types of eating disturbance; “Appetite change” was shown in nearly half of the mild AD patients (49.5%). In the moderate stage, the scores of “change of eating habits and food preference” were highest, and in the severe stage “swallowing disturbance” became critical.

**Conclusion:**

In AD, the relationship of dementia stage to eating disturbance differs according to the type of eating disturbance. The relationships between various eating disturbance and the severity of dementia should be considered.

## Introduction

Eating is essential to life and is one of the most important daily activities for managing patients with dementia. In caring for patients with dementia, eating takes up as large a share as help with bathing and toilet support. It is well known that various eating disturbance occur with dementia progression, including “swallowing disturbance”, “change of appetite”, “change of eating habits”, “consumption of inedible objects” and so on. These symptoms are thought to be modulated by many factors including cognitive dysfunction, psychiatric and neurological symptoms, and decline of daily activity in individuals with dementia [1,2,3,4,5]. Despite the importance of the disturbance, there have been few systematic studies of eating disturbance compared with the other behavioral and psychological symptoms of dementia (BPSD) like hallucination and delusions.

Several studies have reported that different types of dementia present with characteristic behavioral profiles reflecting the specific brain regions affected. Furthermore, recent studies have found that the features of BPSD might be influenced by dementia stage [6,7,8]. With regard to eating and swallowing disturbance, characteristics of the symptoms in each dementia are getting clear. Patients with Alzheimer’s disease (AD) sometimes suffer loss of appetite and decrease their body weight [9,10]. Some patients with vascular dementia (VaD) have pseudobulbar palsy resulting in difficulty swallowing and have a high risk of aspiration pneumonia [11,12,13,14]. Patients with dementia with Lewy bodies (DLB) have difficulty swallowing and loss of appetite [4]. Patients with frontotemporal dementia (FTD) and semantic dementia (SD) increase in appetite, come to prefer sweet and strong foods, and want to eat the same foods repeatedly [3,15]. However, most of the studies focused on a part of eating disturbance such as swallowing and appetite. There have been few comprehensive studies including eating habits and food preference in patients with AD.

The aims of this study were to investigate almost all eating disturbance and to reveal the relationship between dementia stage and characteristics of eating disturbance in patients with AD.

## Materials and Methods

### Ethics Statement

Before the study, a research plan was composed and put forward to the Ethics Committee of Kumamoto University School of Medicine, which was a typical comprehensive university in Japan, for reviewal and approval. All procedures for the present study strictly followed the 2011 Clinical Study Guidelines of the Ethics Committee of Kumamoto University Hospital and were approved by the Internal Review Board. After a complete description of the study was presented, informed written consent was obtained from patients and their caregivers in compliance with the research standards for human research for all participating institutions and in accordance with the Helsinki Declaration.

### Subjects

This study was a prospective hospital-based cohort study. A total of 220 outpatients were selected according to the following inclusion/exclusion criteria from a consecutive series of 407 patients with dementia who attended the memory clinic of Nozaki Hospital, which is a psychiatric hospital, from April 2012 to June 2013. Among them, 60 were men and 160 women. All individuals were examined by senior neuropsychiatrists (K.K. and K.A.) using routine laboratory tests, standard neuropsychological examinations such as Mini-Mental State Examination (MMSE) [16] and brain magnetic resonance imaging (MRI) or brain computed tomography (CT). The inclusion criterion in the present study was fulfilling the National Institute for Neurological and Communicative Disorders and Stroke and the Alzheimer’s Disease and Related Disorders Association for probable AD [17]. The following patients were excluded from the current study: (1) those without a reliable informant; (2) those with developmental abnormalities, serious psychiatric diseases such as schizophrenia and major depression, or substance abuse before the onset of dementia, or significant neurologic antecedents, such as brain trauma, brain tumor, epilepsy, and inflammatory disease; (3) those complicated by an unstable physical illness, such as diabetes mellitus or malignant diseases, that would influence their eating behavior, and (4) those unable to provide informed consent.

Thirty normal elderly subjects (NE subjects) age-matched to patients were recruited from the community (12 male and 18 female). They showed normal cognitive functions (25 or above on the MMSE), normal findings in the physical and neurologic examinations, no history of psychiatric disorders, and no risk factors for cerebrovascular disease (hypertension, heart disease, and diabetes mellitus).

### Measurement

The severity of dementia was rated by using the Clinical Dementia Rating (CDR) [18], of which the scores range between 0 (no cognitive decline), 0.5 (questionable dementia), 1 (mild dementia), 2 (moderate dementia) and 3 (severe dementia). In this study, CDR 0.5 and CDR 1 patients were assigned to one group during the analysis and we considered these patients as having mild dementia. Thus, we compared eating problems between four groups; “mild dementia (CDR 0.5 and 1, n = 99)”, “moderate dementia (CDR 2, n = 63)”, “severe dementia (CDR 3, n = 58)”, and NE subjects (n = 30) groups.

To assess the characteristics of eating disturbance of AD patients, we used a comprehensive questionnaire that had been originally designed to assess eating disturbance in patients with dementia [3]. This caregiver-based questionnaire consisted of 37 items investigating the following five domains: 1) swallowing, 2) appetite, 3) food preference including sweet food preference and food fads, 4) eating habits including stereotypic eating behaviors and decline in table manners, and 5) other oral behaviors including food cramming and indiscriminate eating (Table 1). It was emphasized that a “symptom” should reflect a substantive change from a patient’s premorbid state and evaluated states for the latest month. If caregivers endorsed a particular item, they were asked to rate the frequency (1 = occasionally, less than once per week; 2 = often, about once per week; 3 = frequently, several times per week but less than every day; 4 = very frequently, once or more per day or continuously); and severity (1 = mild, easily controlled; 2 = moderate, not easily controlled; 3 = marked, embarrassing or otherwise disturbing to the family), to derive a product scores (frequency × severity). The questionnaire was administered by a single rater (K.K.).

**Table 1:** Symptoms of eating/swallowing disturbance for each domain in the questionnaire.

**Swallowing disturbance**	Difficulty in swallowing food
	Difficulty in swallowing liquids
	Coughing or choking when swallowing
	Taking a long time to swallow
	Placing food in mouth but not chewing it
	Chewing food but not swallowing it
**Appetite change**	Loss of appetite
	Increase in appetite
	Seeking out food between meals
	Overeating at meal time
	Requesting more food
	Reporting hunger
	Reporting being overfull
	Other change about appetite
	Needs to limit food
**Food preference**	Preferring sweet foods more than before
	Drinking more soft or sweet drinks
	Drinking more tea/coffee or water
	“Taste” in food changed in some way
	Adding more seasoning to their food
	Developing other food fads
	Hoarding foods
	Drinking more alcohol
**Eating habits**	Wanting to cook or eat the same food every day
	Tending to eat foods in the same order
	Wanting to eat at the same time every day
	Decline in table manners
	Eating with hands
	Other change about food preference
	Taking a long time to eat
**Other eating behaviors**	Tending to overfill mouth
	Chewing or sucking without trying to eat
	Eating non-edible foodstuffs
	Tending to snatch or grasp any food items
	Becoming a heavier smoker or taking up smoking
	Episodes of vomiting naturally
	Episodes of vomiting by using their fingers

### Statistical analyses

To compare the rates of each domain (swallowing, appetite, food preference, eating habits, and other oral behaviors) between the 4 groups, we used χ^2^-test with Fisher’s exact probability test and residual analysis using Bonferroni z-test for each comparison when an overall group difference was significant. If a patient had any symptom, we regarded positive in that domain. In the domain of “change of appetite”, “loss of appetite” and “increase in appetite” were analyzed separately because of the possibility of different neural bases of the two symptoms [19,20]. Additionally, the score (frequency × severity) of each domain represented by the total score within each domain was compared between the four groups using one-way analysis of variance (ANOVA) followed by a post-hoc Sidak test. A significance level of 0.05 was set for all analyses. All statistical analyses were performed using IBM SPSS Statistics 21 (IBM Japan, Tokyo, Japan).

## Results

**Fig 1:**
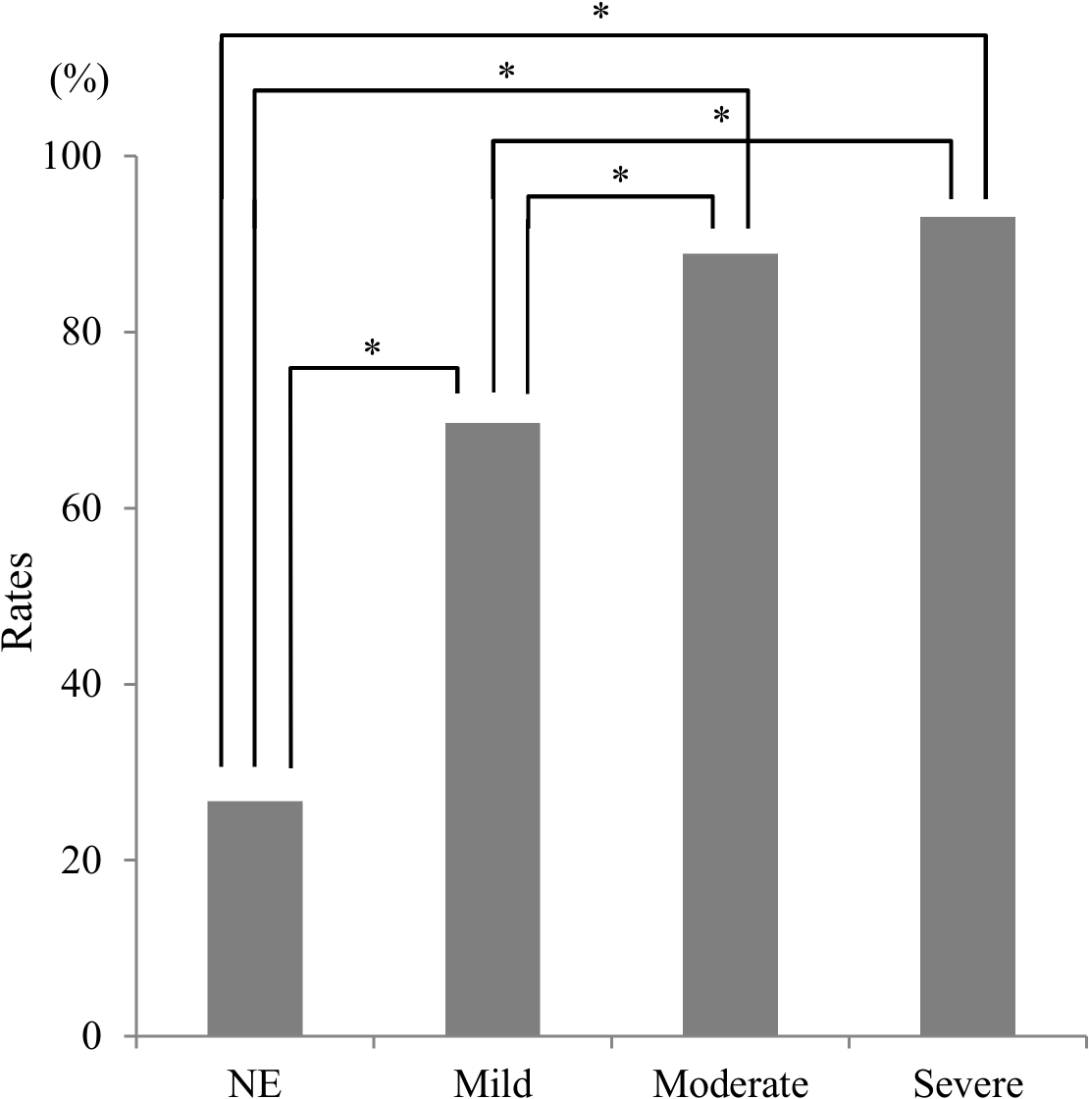
Rates of eating and swallowing disturbance in each stage of dementia and NE subjects. NE: normal elderly; *: An asterisk shows *p* < 0.05 with Bonfferoni correction.

Demographic variables of the four groups are summarized in Table 2. There was a significant difference in sex and age. Of the 220 AD patients, 179 (81.4%) patients showed some eating disturbance. Fig 1 shows the rates of eating disturbance in each of the four groups.

**Table 2:** Demographics of participants.

	NE	MILD	MODERATE	SEVERE		
	SUBJECTS	(CDR 0.5 & 1)	(CDR 2)	(CDR 3)	All	P-VALUE
	(n = 30)	(n = 99)	(n = 63)	(n = 58)	(n = 220)	
**Male/female**	12 / 18	28 / 71	15 / 48	17 / 41	60 / 160	0.458 ^a^
**Age (years)**	80.1 ± 5.0	80.4 ± 6.4	82.1 ± 5.7	83.1 ± 5.6	81.6 ± 6.1	0.019 ^b^
**MMSE**	27.3 ± 2.0	21.6 ± 2.8	16.7 ± 4.1	9.9 ± 5.6	17.1 ± 6.3	<0.001 ^b^
**Disease duration**		2.5±1.9	4.7±2.8	7.1±3.8	4.3±3.4	

Notes: Values are n or mean ± SD.

NE: normal elderly; MMSE: Mini-Mental State Examination; CDR: Clinical Dementia Rating.

^a2^test

^b^ANOVA

Table 3 shows the rates of each eating domain in the three patient groups and in NE subjects. Significant group differences were observed for the rates of all domains except for “loss of appetite”(*p* = 0.153). Z tests showed that the rates of “appetite change”(*p*<0.001), “food preference”(*p*<0.001) and “eating habit”(*p*<0.001) domains was significantly higher in the three patient groups than in the NE subjects group. The rates of “swallowing disturbance” and “other eating behaviors” domains were significantly higher in the severe group than in any other group.

**Table 3:** Rates of disturbance in each domain for the subject groups.

	NE	MILD	MODERATE	SEVERE		
	SUBJECTS	(CDR 0.5 & 1)	(CDR 2)	(CDR 3)	P-VALUE	Z TEST WITH BONFERRONI CORRECTION
	(n = 30)	(n = 99)	(n = 63)	(n = 58)		
**Swallowing disturbance**	16.7%	22.2%	28.6%	53.4%	< 0.001	NE, Mild, Moderate < Severe
**Appetite change**	13.3%	49.5%	58.7%	77.6%	< 0.001	NE < Mild, Moderate, Severe; Mild < Severe
** Loss of appetite**	13.3%	28.3%	22.2%	34.5%	0.153	
** Increase in appetite**	0.0%	27.3%	39.7%	48.3%	< 0.001	NE < Mild, Moderate, Severe; Mild < Severe
**Food preference**	6.7%	36.4%	47.6%	46.6%	< 0.001	NE < Mild, Moderate, Severe
**Eating habits**	0.0%	38.4%	60.3%	58.6%	< 0.001	NE < Mild, Moderate, Severe; Mild <Moderate
**Other eating behaviors**	0.0%	9.1%	15.9%	37.9%	< 0.001	NE, Mild, Moderate < Severe

Note: NE: normal elderly; CDR: Clinical Dementia Rating.

Table 4 shows the score of each eating domain. The score of all eating domains except for the “loss of appetite” increased with dementia severity, similar to the rates. It was noteworthy that the score of the “Food preference” domain in the moderate stage was significantly higher (p = 0.002) than in the severe one.

**Table 4:** Scores for each eating/swallowing domain in the four subject groups.

	NE	MILD	MODERATE	SEVERE		
	SUBJECTS	(CDR 0.5 & 1)	(CDR 2)	(CDR 3)	P-VALUE	POST HOC SIDAK TEST
	(n = 30)	(n = 99)	(n = 63)	(n = 58)		
**Swallowing disturbance**	0.17 ± 0.4	1.36 ± 6.5	2.98 ± 9.8	5.22 ± 8.8	0.007	NE, Mild < Severe
**Appetite change**	0.13 ± 0.4	3.24 ± 4.7	7.10 ± 12.7	5.71 ± 7.6	< 0.001	NE < Moderate, Severe; Mild < Moderate
** Loss of appetite**	0.13 ± 0.4	1.53 ± 3.1	1.52 ± 4.0	2.17 ± 3.9	0.069	
** Increase in appetite**	0.00 ± 0.0	1.73 ± 4.1	5.57 ± 12.1	3.53 ± 7.4	0.002	NE, Mild < Moderate
**Food preference**	0.07 ± 0.3	2.31 ± 5.3	5.10 ± 10.1	2.00 ± 3.6	0.002	NE, Mild < Moderate; Severe < Moderate
**Eating habits**	0.00 ± 0.0	2.30 ± 4.6	6.44 ± 9.4	4.81 ± 6.4	< 0.001	NE < Moderate, Severe; Mild < Moderate
**Other eating behaviors**	0.00 ± 0.0	1.34 ± 0.7	0.70 ± 2.34	1.66 ± 3.2	< 0.001	NE, Mild < Severe

Notes: Values are mean ± SD.

NE: normal elderly; CDR: Clinical Dementia Rating.

## Discussion

In this study, we examined eating disturbance in a large (n = 220) sample of patients with AD. To our knowledge, this is the first study that used a single, previously validated questionnaire to evaluate eating disturbance in AD in relation to dementia severity.

The most remarkable finding was the fact that as many as 81.4% patients with AD showed some eating disturbance, whereas only 26.7% of the NE subjects had disturbance. The rates of eating disturbance, assessed by using Neuropsychiatric Inventory (NPI), in patients with AD was previously reported as 23.5% to 51.6% [8,21]. The higher rates of our study might be due to the sensitivity of our assessment tool. The large difference in rates between patients with AD and the NE subjects demonstrates the significance of eating disturbance for managing patients with AD.

Previously, it was reported that the rates of all symptoms in NPI increased along the severity of the disease, except for sleep and eating disturbance [22]. They showed that the rates of “eating disturbance” was 24.5% in moderate-severe AD, 15.2% in mild AD, 19.8% in Mild Cognitive Impairment, and 6% in controls and no significant differences were found among the groups. In the present study, “increase in appetite”, “swallowing disturbance” and “other eating behaviors” increased in proportion to severity of dementia. On the other hand, the score of “food preference” and “eating habits” became highest in the moderate stage and declined afterward. The relationship of dementia stage should be different among the domains of eating disturbance.

Patients with AD in the mild stage have been reported to show few eating disturbance, similar to the neurologic manifestations [3,4,5,15]. However, in the present study, patients with AD had many types of eating disturbance even in an early stage. Especially, “appetite change” was shown in nearly half of the mild patients with AD (49.5%). The difference might be partially explained by sampling bias. Subjects in the previous studies were from the memory clinic in a university hospital while those in this study were from the memory clinic in a psychiatric hospital. Weight loss and low body weight have been commonly reported in AD. Furthermore, a recent longitudinal study, involving community-dwelling elderly, found that participants with incident dementia or MCI had accelerated weight loss from as early as 6 years before diagnosis of AD [23]. In the present study the rates of “loss of appetite” was about twice as high in each severity group of AD as in the NE subjects group although the difference between patients with AD and NE subjects did not reach the significance level. We should consider the importance of “loss of appetite” in all severity stages of AD patients. It was interesting that two conflicting eating symptoms, “increase in appetite” and “loss of appetite”, were observed in approximately the same number of patients with mild AD. Although fundamental pathophysiological mechanism for “appetite change” is unclear, this condition might reflect different neuropsychological and neuropsychiatric symptoms associated with AD. Appetite loss is well known as being a main symptom of depression and as many as 68.0% patients with AD were reported to have some depressive symptoms [24]. There was a possibility that depression might be concerned about “loss of appetite”. On the other hand, “increase in appetite” might reflect the behavior of having a meal in a repetitive manner because of severe memory impairment. We need to do a longitudinal study for revealing whether a case of “loss of appetite” convers to a case of “increase in appetite”.

In the mild stage, over one-third of patients showed changes in “food preference” and “eating habits”. The score of “food preference”, “eating habits”, “appetite change” and “increase in appetite” became highest in the moderate stage and declined afterward even if some are not statistically significant. It may reflect some sort of ‘burnout’ leading to increased behavioral apathy. These eating behaviors may require a certain level of functional ability in patients with AD. The details of “food preference” were changing to prefer sweet foods and candies, and adding strong flavor to their dishes using soy sauces. It is not clear yet whether the gustatory function is impaired in patients with AD. Previous research reported that the gustatory function of patients with AD was not impaired [25,26]. On the other hand, Steinbach et al. (2010) investigated the gustatory recognition threshold in mild AD patients, MCI patients, and normal elderly subjects using an actual taste test, and they showed the impairment of gustatory recognition for all four basic tastes in the patient group, even at the MCI stage [27]. Although the present study did not make a direct examination of gustatory function of patients with AD, our results may indicate the possible presence of gustatory dysfunction in mild AD in an indirect fashion.

“Swallowing disturbance” was critical in the severe stage AD. Humbert et al. investigated the relationship between swallowing disturbance and severity of dementia, and they found that there were a few patients with AD who showed swallowing disturbance in their mild or moderate stage, and more patients presented with this symptom as dementia progressed [28]. In the current study, approximately half of the patients in the severe stage had developed swallowing disturbance, which was lower than we expected. We thought that this might be due to the fact that some of our patients with swallowing disturbance had already been taken care by professional caregivers or the nursing staff in a daycare unit using a thick liquid and minced food.

There are a few methodological issues that should be taken into consideration when interpreting our results. First, the diagnosis relied solely on clinical basis without histopathologic confirmation, with inevitably some uncertainty about the rate of misclassification. Secondly, we did not consider the influence of other neuropsychiatric symptoms and neuropsychotropic drugs such as neuroleptics, SSRIs, SNRIs, and cholinesterase inhibitors. These symptoms and drugs might have some effects of changing appetite and swallowing disturbance. Thirdly, due to the cross-sectional study design, the relationship between the severity of dementia and eating disturbance could only be hypothesized. It remains an open question whether “loss of appetite” and “increase in appetite” shift relative to each other in the same patient with disease progression or if the two conditions are shown in different patients. Longitudinal research may offer additional information on the development and course of eating disturbance in patients with AD.

## Conclusion

The present findings indicated the importance of paying attention to various eating disturbance corresponding to the severity of dementia. With foresight, caregivers can handle eating disturbance in AD when the characteristics and longitudinal changes are revealed.

## Supporting Information

S1 Table Data of this study including values and statistical results. (XLSX)
